# Transcriptomic and Proteostasis Networks of CFTR and the Development of Small Molecule Modulators for the Treatment of Cystic Fibrosis Lung Disease

**DOI:** 10.3390/genes11050546

**Published:** 2020-05-13

**Authors:** Matthew D. Strub, Paul B. McCray, Jr.

**Affiliations:** 1 Interdisciplinary Graduate Program in Genetics, The University of Iowa, Iowa City, IA 52242, USA; matthew-strub@uiowa.edu; 2 Stead Family Department of Pediatrics, The University of Iowa, Iowa City, IA 52242, USA

**Keywords:** Cystic fibrosis, CFTR, transcriptomics, proteostasis, small molecules, drug development

## Abstract

Cystic fibrosis (CF) is a lethal autosomal recessive disease caused by mutations in the CF transmembrane conductance regulator (*CFTR*) gene. The diversity of mutations and the multiple ways by which the protein is affected present challenges for therapeutic development. The observation that the Phe508del-CFTR mutant protein is temperature sensitive provided proof of principle that mutant CFTR could escape proteosomal degradation and retain partial function. Several specific protein interactors and quality control checkpoints encountered by CFTR during its proteostasis have been investigated for therapeutic purposes, but remain incompletely understood. Furthermore, pharmacological manipulation of many CFTR interactors has not been thoroughly investigated for the rescue of Phe508del-CFTR. However, high-throughput screening technologies helped identify several small molecule modulators that rescue CFTR from proteosomal degradation and restore partial function to the protein. Here, we discuss the current state of CFTR transcriptomic and biogenesis research and small molecule therapy development. We also review recent progress in CFTR proteostasis modulators and discuss how such treatments could complement current FDA-approved small molecules.

## 1. Cystic Fibrosis

Cystic Fibrosis (CF) is the most common lethal autosomal recessive disease in Caucasian populations, with approximately 75,000 individuals worldwide suffering from the condition [1,2]. In the United States, CF was first identified as a clinical syndrome in 1938 by Dr. Dorothy Andersen, who observed fluid-filled cysts and scars within the pancreas and similar tissue damage in the lungs of deceased children that had experienced digestive and respiratory problems [3]. Dr. Andersen termed the disease “cystic fibrosis of the pancreas”. CF negatively affects multiple organ systems and can cause meconium ileus, cholestasis, biliary cirrhosis, increased sweat chloride concentrations, infertility, diabetes, and growth failure, among other symptoms [4,5,6,7,8,9,10,11]. However, the majority of morbidity and mortality associated with CF results from chronic and progressive lung dysfunction, characterized by altered airway surface liquid pH, decreased host defenses at the airway surface, impaired mucociliary transport resulting in chronic bacterial infections, bronchiectasis, irreversible tissue remodeling, and respiratory failure. Following the identification of elevated levels of chloride in the sweat of CF patients, it was hypothesized that the sweat ducts of these patients were impermeable to chloride [12]. Subsequent patch-clamp analyses of nasal and airway epithelial cells confirmed the defect in chloride permeability of the plasma membranes [13,14,15,16]. In 1989 Choi, Collins, and colleagues used linkage-based techniques to identify the gene responsible for CF, which they named the *Cystic Fibrosis Transmembrane conductance Regulator* (*CFTR*) [17,18,19].

## 2. CFTR Mutation Classes

**Figure 1:**
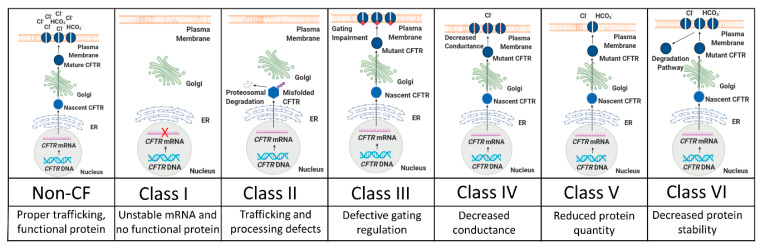
Schematic representation of CFTR (CF transmembrane conductance regulator) mutation classes. The top panels briefly illustrate CFTR trafficking along its proteostasis pathway and how protein maturation is disrupted by mutations. The middle panels list the mutation class, while the bottom panels briefly describe the defect(s) associated with each class. Adapted from [28].

The *CFTR* gene is on the long end of chromosome 7 and approximately 180,000 base pairs in length. *CFTR* is a member of the superfamily of ATP-binding cassette (ABC) genes and encodes an anion channel that conducts chloride, bicarbonate, and other substrates, thereby regulating the composition and volume of epithelial secretions [20,21,22,23,24]. To date, over 2,000 unique mutations have been identified in *CFTR*, resulting in an extensive range of disease severity [25]. These mutations have been grouped into six different classes based on the mechanisms by which they are believed to alter CFTR expression and function (Figure 1) [26,27]. Individual mutations may negatively affect CFTR function by more than one mechanism, and therefore, fall into multiple classes.

### 2.1. Class I Mutations: Unstable mRNA and No Protein Production

Approximately 5–10% of *CFTR* mutations are associated with protein production, resulting from unstable mRNA and little or no CFTR protein [29]. These mutations can be caused by insertion/deletion frameshifts, abnormal splicing, or premature stop codons [30]. Examples of Class I mutations include R553X and G542X, the second most common *CFTR* mutation.

### 2.2. Class II Mutations: Trafficking and Processing Defects

Defective protein processing occurs in the most common class of *CFTR* mutations. Class II proteins fail to traffic through the CFTR proteostasis pathway and rarely arrive at the cell membrane [31,32]. Phe508del, the most common CF-causing mutation, is caused by a three base pair deletion (Δ) on exon 11 that results in the loss of a phenylalanine at residue 508 (Phe508del). Phe508del accounts for an estimated 70% of mutant *CFTR* alleles in the United States, and thus, roughly 90% of CF patients have one or two Phe508del alleles [33]. Class II mutant proteins often fail to reach the Golgi apparatus and are therefore never fully glycosylated [34]. Instead, these proteins are identified as misfolded by endoplasmic reticulum-associated protein degradation (ERAD) quality control mechanisms and are subsequently degraded [26]. As a result, Class II proteins rarely reach the cell surface to function [34]. Importantly, if Class II proteins do reach the cell surface, partial function can occur, although membrane stability is often impaired following rescue [31,35].

### 2.3. Class III Mutations: Gating Impairments

Mutations in the nucleotide binding domains (NBD) or phosphorylation sites of the regulatory domain of *CFTR* can cause reduced channel activity [26,36,37]. The third most common *CFTR* mutation, G551D, produces a protein that, despite reaching the cell membrane, has approximately 100-fold lower open probability than that of wild-type (wt) [38]. Dimerization of the NBDs of CFTR forms two ATP binding pockets, termed ABP1 and ABP2 [39]. Whereas binding of ATP to ABP1 helps to stabilize the open channel conformation of CFTR, channel opening is dependent on ATP binding of ABP2 [40]. The G551D mutation, located in ABP2, prevents ATP binding, thus inhibiting opening of the CFTR channel [38]. The S1255P mutation, found in NBD2, also does not disrupt CFTR maturation, but instead alters the ATP-binding pocket, resulting in gating instability [41].

### 2.4. Class IV Mutations: Decreased Conductance

Class IV proteins achieve proper processing and gating, but mutations in their membrane spanning domains cause a misshapen protein that restricts anion transport [26]. This results in a decreased rate of ion flow through each open channel and an overall decrease in current conducted by CFTR [42]. Mutations affecting channel pore activity often arise in arginine residues (e.g., R117H, R347P, R334W). Some Class IV mutations, including R117H, also decrease the open probability of CFTR [43].

### 2.5. Class V Mutations: Reduced Protein Quantity

Inefficient protein maturation can be caused by alternative splicing, amino acid substitutions, or promoter mutations [26]. Class V mutations often produce incorrectly spliced versions of the *CFTR* mRNA in variable proportions, and the resultant proteins rarely transit to the cell membrane, resulting in a decreased number of functioning CFTR channels [44,45]. The most prevalent examples of Class V mutations include c.3717+12191C>T and c.3140-26A>G [46,47].

### 2.6. Class VI mutations: Unstable Protein

Class VI mutants can act as functional proteins at the cell surface. However, instability in the protein structure results in reduced residency at the cell surface, more rapid protein turnover, and therefore, less ion conductance [33,45]. Examples include c.120del123 and Phe508del when rescued by low temperature or correctors (rPhe508del) [48].

## 3. CFTR Structure and Function

**Figure 2:**
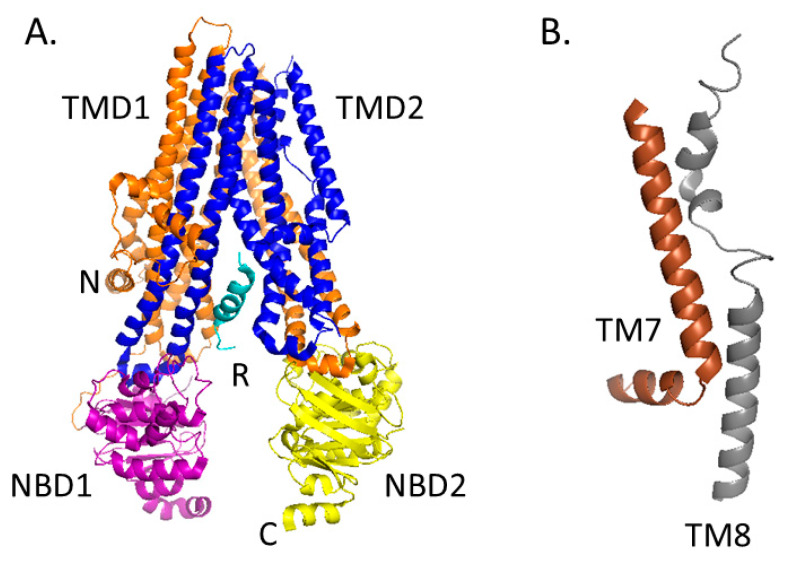
Cryo-EM structure of dephosphorylated, ATP-free CFTR. **A.** CFTR contains two transmembrane domains (TMD1 in orange, TMD2 in blue), two nucleotide binding domains (NBD1 in purple, NBD2 in yellow), and a regulatory (R) domain (cyan). CFTR is activated by phosphorylation of the R domain and ATP hydrolysis by the NBDs. Note that the structural flexibility of the R domain limits its visibility by Cryo-EM. Instead, 19 alanines are shown that correspond to the C-terminal region of the R domain. **B.** Magnified view of transmembrane helices (TM) 7 (brown) and 8 (gray). CFTR differs from other ABC transporters in that TM7 is displaced from its usual position and TM8 breaks into three short helices, rather than being a continuous helix as seen in other ABC transporters. TM7 and TM8 are found in TMD2. PBD ID: 5UAK.

CFTR is a 1,480 amino acid transmembrane glycoprotein containing two homologous halves, each consisting of six transmembrane alpha helices (termed TMD or transmembrane domain) that form an anion conduction pore, and a nucleotide-binding domain that serves as the binding site for ATP hydrolysis (Figure 2). These halves are connected by a regulatory (R) domain that contains multiple phosphorylation sites and regulates channel activity. The R domain is intrinsically unstructured and adapts its conformation upon binding to the NBDs and the CFTR N-terminus [49,50]. Recently, the cryo-EM structure of full length human CFTR was published, highlighting several key structural elements required for a fully functional protein [51,52]. First, an unphosphorylated R domain prevents the dimerization of NBD1 and NBD2, resulting in a closed channel. Secondly, a small inhibitory helix exists in the R domain that is docked inside the intracellular vestibule between the nucleotide binding domains, which precludes channel opening. It is believed that the disruption of the interaction between this inhibitory helix and the nucleotide binding domains would allow for protein kinase A (PKA)-mediated phosphorylation of the R domain, resulting in NBD dimerization and subsequent opening of the ion channel. Third, the authors sought to explain why CFTR acts as an ion channel, whereas other ABC transporters function as pumps that move ions against electrochemical gradients. When comparing the structures of CFTR and other ABC proteins, differences in two transmembrane helices (TM7 and TM8) were identified, leading to the hypothesis that these helices affect ion conduction and gating.

CFTR is neither isolated from neighboring proteins nor does it act alone. Instead, CFTR is part of a multiprotein assembly at the apical membrane surface, and is anchored via PDZ domains commonly found in plasma membrane proteins and other intracellular signaling proteins [53,54]. When at the apical membrane, CFTR is spatially located near other ion channels, membrane receptors, and cytoskeletal proteins. Additionally, transcriptomic studies in human CF cells suggest that significant changes in gene expression may result from the absence of functional CFTR [55]. Affected genes include members of the protein processing and inflammatory response functional families, among others. Likewise, proteomic analyses of the wt and Phe508del-CFTR interactomes by Pankow et al. identified novel effectors belonging to mRNA decay, co-translational control, endocytic recycling, ER quality control and folding, and protein degradation networks [56]. Such relationships between *CFTR* and other genes suggest that CFTR does not act solely as an ion channel, but instead, may have various roles throughout its biogenesis. Interactions between *CFTR* and genes that influence its processing or maturation could help to explain the wide range of phenotypes and severities in CF patients with identical mutations [33]. A better understanding of such interactions could also lead to the development of small molecule modulators for CF lung disease.

## 4. Cystic Fibrosis Transcriptome

### 4.1. mRNA Profiling

**Figure 3:**
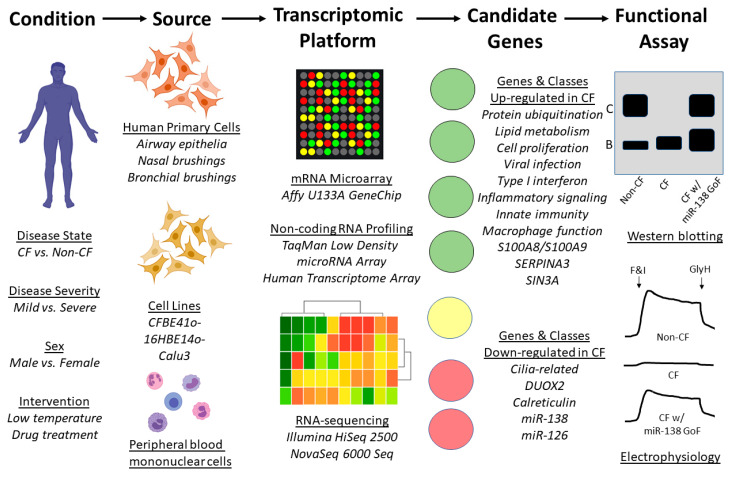
Workflow of transcriptomic profiling. To identify transcriptomic changes resulting from cystic fibrosis (e.g., disease presence or severity), multiple primary and immortalized cell sources are available, as are several profiling platforms. Analysis of profiling output reveals differentially expressed genes (DEGs) and gene classes; findings highlighted in the text are shown under “Candidate Genes”. The effects of manipulating DEGs (e.g., gain of function (GoF) can be assessed using multiple assays. Examples of CFTR western blotting and electrophysiology as endpoints are presented with hypothetical data representing overexpression of miR-138 [58]. F&I represent the cyclic AMP agonists forskolin and IBMX. GlyH represents CFTR inhibitor GlyH-101.

The first transcriptomic profiling of well-differentiated primary cultures of human airway epithelia from Phe508del/Phe508del donors was performed by Zabner et al. in 2005 [57]. Of the approximately 22,000 genes represented on the Affymetrix U133A GeneChip, 18 were observed to be significantly upregulated in CF, while 6 were downregulated. The KCl cotransporter *KCC4* was identified as elevated in CF and was deemed a candidate for further studies. Interestingly, this profiling concluded that the level of *CFTR* mRNA was not significantly different in Phe508del/Phe508del cells compared to non-CF. Differences were also not observed when comparing cells from male and female donors. Figure 3 shows a general workflow of transcriptomic profiling for CF.

One of the earliest transcriptomic studies using nasal epithelium of CF subjects was performed by Wright et al., in which they compared Phe508del homozygotes in the most severe 20th percentile of lung disease (as measured by forced expiratory volume; FEV_1_) to those in the mildest 20th percentile [59]. Phe508del homozygotes and age-matched non-CF controls were also compared. Significant upregulation of 569 genes was observed in severe CF lung disease, while genes involved in protein ubiquitination (discussed later in Phe508del-CFTR Proteostasis and Quality Control), mitochondrial oxidoreductase activity, and lipid metabolism were significantly enriched. Among genes downregulated in CF were *DUOX2*, a key producer of hydrogen peroxide for airway mucosal defense, and calreticulin, an ER chaperone involved in protein metabolism (also discussed later in Phe508del-CFTR Proteostasis and Quality Control). Genes upregulated in mild CF lung disease compared to severe CF and non-CF included statherin, which is known to be produced in the submucosal cavities of the upper airways and to have antibacterial properties, and *ADIPOQ*, an anti-inflammatory cytokine and inducer of IL-10. RT-PCR revealed no significant differences in the transcriptomic levels of *CFTR* between Phe508del homozygotes and non-CF controls, in agreement with the findings by Zabner et al. [57].

Clarke and colleagues carried out a whole genome microarray study of primary nasal epithelial cells from Phe508del homozygotes and non-CF controls, and compared their results with several other relevant microarray datasets [60]. In their expression profile, genes involved in cell proliferation were significantly upregulated in CF, while cilia-related genes were downregulated. Due to great variability in the gene expression profiles across the independent studies, the meta-analysis comparing this study to five other microarray experiments (including the Zabner, Wright, and Ogilvie studies described in this section) yielded few common dysregulated genes across at least three experiments. However, when the authors compared their microarray results with the Ogilvie microarray, a molecular signature of native CF airway epithelial cells was observed, consisting of 21 common upregulated genes and 9 common downregulated genes [61]. A significant number of these genes were involved in inflammation and defense, including the upregulated *CXCR4, FOS, S100A8, S100A9,* and *SERPINA3* transcripts.

Although several gene expression studies have used nasal epithelial brushings from CF donors, Ogilvie et al. concluded that transcriptomics of the CF nasal epithelium is not representative of gene expression in the lung respiratory epithelium. Following bead array profiling of CF and non-CF nasal and bronchial epithelium, 863 genes were found to be significantly dysregulated in the bronchial cells, whereas only 15 genes were identified as dysregulated in nasal cells [61].

Polineni et al. performed RNA-sequencing of nasal mucosal cells from 134 CF subjects with varying genotypes and disease severities, as assessed by cytokine levels in nasal lavages [62]. Pathway analysis of the gene expression data highlighted the positive correlation between CF disease severity and viral infection, inflammatory signaling, lipid metabolism, macrophage function, and innate immunity. Multiple human leukocyte antigen (HLA) genes robustly contributed to the enriched pathways and several were also observed at the intersection of the gene expression profiling and previously identified CF GWAS risk alleles. The authors concluded that HLA genes may serve as targets for interventions aiming to improve CF lung health.

A meta-analysis of 13 microarray experiments was performed by Clarke and colleagues, comparing CF with similar disorders (e.g., chronic obstructive pulmonary disease, asthma, and idiopathic pulmonary fibrosis), environmental factors (e.g., smoking), relevant cellular processes (e.g., epithelial regeneration), and non-respiratory controls (e.g., schizophrenia) [63]. Genes whose expression was inversely related with *CFTR* across samples expressing Phe508del were subjected to an siRNA knockdown assay to identify potential negative regulators of CFTR. Nine genes, including *SNX6*, *PSEN1*, and *RCN2,* produced an appreciable increase in CFTR trafficking to the cell membrane. While the siRNA knockdown experiments were considered preliminary by the authors, these genes may serve as intriguing leads for therapeutic targets.

An additional transcriptomic study comparing the peripheral blood leukocytes of CF subjects with mild and severe lung disease was performed by Kormann et al. [64] Enrichment analyses identified genes of the type I interferon response, as well as ribosomal stalk proteins, as upregulated in mild disease. Such modifiers of CF lung disease may have implications as new biomarkers or targets for intervention.

### 4.2. Non-coding RNA Profiling

While most CF-related transcriptomic studies have focused on mRNA profiling, McCray and colleagues profiled global microRNA expression in well-differentiated primary cultures of human airway epithelia by qPCR and identified 31 highly expressed microRNAs in CF [58]. Further analyses of these microRNAs identified *SIN3A* as a highly conserved target of miR-138. As *SIN3A* has conserved motifs that bind to the transcriptional repressor CTCF and the *CFTR* locus contains functional CTCF-binding sites, the authors hypothesized that miR-138 and *SIN3A* regulate *CFTR*. Functional assays determined that overexpression of miR-138 or knockdown of *SIN3A* partially restored the maturation, trafficking, and function of Phe508del-CFTR. Oglesby et al. also identified miR-126 as downregulated in CF airway epithelial cells [65]. Overexpression of miR-126 resulted in downregulated TOM1 protein production. Furthermore, knockdown of *TOM1* mRNA significantly increased NF-κB regulated IL-8 secretion, linking miR-126 to innate immune responses in CF. Additionally, miR-145 has been shown to mediate TGF-β inhibition of CFTR function and knockdown of miR-145 restored Phe508del function in human primary epithelial cells [66]. Likewise, miR-200b reduces CFTR during prolonged hypoxia, although inhibition of miR-200b also rescues *CFTR* mRNA levels in primary bronchial epithelial cells [67]. These studies lend further support to the strategy of manipulating microRNAs or their target genes to enhance CFTR expression or alleviate symptoms associated with CF.

Likewise, Kamei and colleagues analyzed the expression of non-coding genes, or functional RNAs with no protein-coding capacity [68]. Using the Human Transcriptome Array, 91 dysregulated non-coding RNAs were identified in the CFBE41o- cell line. Linc-SUMF1-2, an intergenic non-coding RNA with no known function, was found to be inversely correlated with wild-type CFTR. Further analyses identified eight dysregulated genes, including *CXCL10*, *MYC*, and *LAMB3*, as both CFTR- and linc-SUMF-1-2-dependent in CF airway epithelial cells, uncovering a novel regulatory pathway of CF-associated gene regulation.

## 5. Phe508del-CFTR Proteostasis and Quality Control

**Figure 4:**
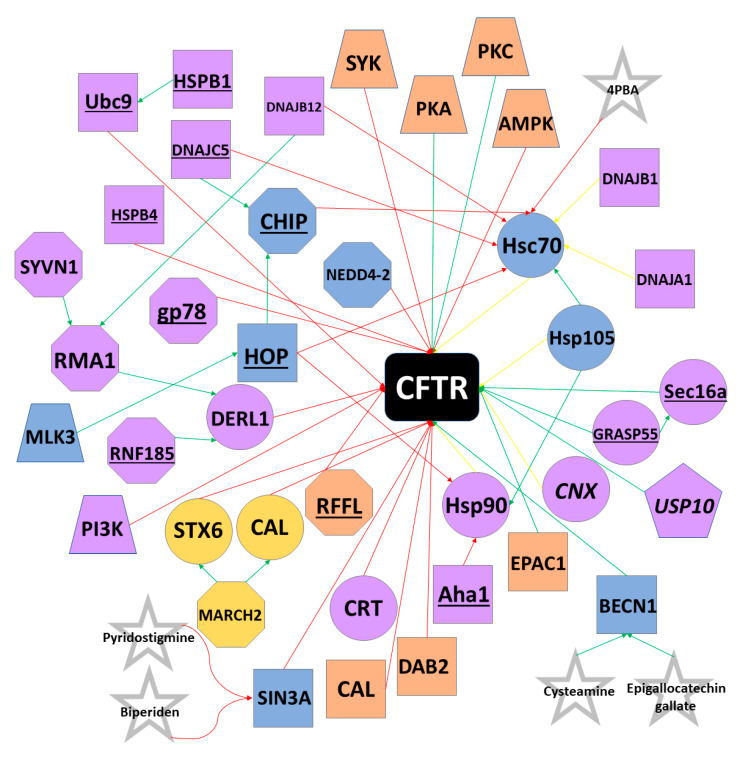
CFTR Proteostasis Interactors. Selected proteins active in the CFTR proteostasis pathway are shown. Octagons represent E3 ubiquitin ligases; trapezoids indicate kinases; circles represent chaperones; pentagons indicate deubiquitinases; co-chaperones and all other proteins are represented as squares; proteostasis modulators are represented as stars. Red arrows indicate degradation interactions; green arrows represent activation or maturation interactions; yellow arrows indicate that the protein can have degradative or activation interactions. In most cases, these proteins degrade Phe508del, while promoting wtCFTR maturation. Proteins shaded in purple primarily interact with co-factors or CFTR at the ER; orange at the cell surface; yellow at the Golgi apparatus. Proteins shaded in blue can interact with co-factors or CFTR at the ER or cell surface. Underlined proteins are primarily only found in the Phe508del proteostasis pathway, whereas italicized proteins are usually found in the wtCFTR pathway. Curved lines indicate that pyridostigmine and biperiden are believed to act by mimicking the transcriptional changes resulting from downregulation of SIN3A. Please note that the most common interactions and locations for each protein are shown. Some proteins are active at multiple locations.

CFTR was among the first membrane proteins identified as being regulated by the ERAD pathway [69]. Wild-type CFTR undergoes co-translation and N-glycosylation in the ER before being packed into COPII vesicles at ER exit sites and trafficked to the Golgi apparatus. Upon reaching the Golgi, glycan processing and modification occurs, rendering a complex, mature form of the protein. CFTR is then trafficked to the cell surface, where its stability is tightly regulated by protein interactors. Upon removal from the plasma membrane, CFTR can undergo endocytosis or be recycled back to the cell surface [70]. Despite its proteostasis pathway being incompletely understood, CFTR interacts with several classes of proteins and must pass multiple quality control checkpoints during trafficking from the ER to the cell surface (Figure 4).

### 5.1. Chaperones and Protein Folding

Heat shock proteins (Hsps) serve as the first quality control constituents of CFTR biogenesis, as these molecular chaperones co-translationally interact directly with CFTR. Hsp90, the constitutively expressed isoform of Hsp70 (often referred to as Hsc70), and the stress-induced isoform of Hsp70 bind CFTR during translation and assist in proper folding via ATP hydrolysis [71,72,73,74,75]. Small molecule-induced inhibition of Hsp90 in cultured human cells prevents proper CFTR folding, leading to protein degradation [71]. However, both Hsp90 and Hsp70 can recruit channel folding and maturation antagonizers, such as the Hsp70/Hsp90 organizing protein (HOP) [76,77]. HOP directs CFTR towards the degradation pathway by recruiting the E3 ubiquitin ligase CHIP (sometimes referred to as STUB1) to the CFTR-Hsp70/90 complex [78,79]. CHIP tags CFTR with ubiquitin (discussed in *E3 Ubiquitin Ligases*) and the CFTR protein is ushered toward the proteasome for degradation. The pro-degradation effects of CHIP can be reversed, however, by the Hsp/Hsc70 nucleotide exchange factor HspBP1, which inhibits CHIP and results in the continuation of CFTR along the pro-folding pathway [78,79]. The Hsp90 co-chaperone Aha1 is believed to prevent Hsp90 from properly interacting with CFTR, resulting in degradation of nascent protein [80,81]. An additional nucleotide exchange factor for Hsc70, Hsp105, has been observed to both promote the post-translational maturation of CFTR, while also at times assisting in the co-translational degradation of CFTR [82,83].

Hsp40 co-chaperones, often referred to as J proteins, have also been shown to interact with CFTR during its initial translation stages. DNAJA1 (Hsp40/Hdj2) and DNAJB1 (Hsp40/Hdj1) interact with Hsc70 to promote folding of the NBD1 of CFTR and assist in rescuing wtCFTR from endoplasmic reticulum retention [73,84,85]. However, these DNAJ proteins have been unable to rescue Phe508del-CFTR from being degraded and sometimes actually serve as pro-degradation components of the quality control machinery [86]. DNAJA1 has been shown to promote CHIP ubiquitin ligase activity and DNAJC5 (Hsp40 cysteine string protein; sometimes referred to as Csp) independently recruits CHIP to the CFTR-Hsp90 complex [87,88,89]. Similarly, DNAJB12 recruits the E3 ubiquitin ligase RMA1 to the CFTR-Hsc70 complex, promoting degradation of both Phe508del-CFTR and immature wtCFTR [90,91].

An additional subclass of Hsps, termed small heat shock proteins (sHsps), have also been shown to affect CFTR biogenesis through holdase activity of misfolded proteins [92]. HSPB1 (sHsp Hsp27) recruits Ubc9 to the immature NBD1 domain, where it catalyzes the attachment of small ubiquitin-like modifier (SUMO), leading to ubiquitination [93,94]. Likewise, HSPB4 (sHsp αA-crystallin) can also assist with CFTR degradation [93].

Calnexin (CNX) is a membrane chaperone found in the ER that affects the folding of CFTR transmembrane domains [95]. While the binding of CNX to CFTR can prevent pro-degradation quality control proteins from binding immaturely folded CFTR, studies also suggest that CNX can obstruct channel maturation [96,97]. In fact, inhibition of CNX improves trafficking of wtCFTR from the ER to the cell membrane [96]. However, such inhibition has little effect on Phe508del-CFTR, perhaps indicating that Phe508del is targeted for degradation prior to the role of CNX in the quality control pathway. Calreticulin (CRT), found in the ER lumen, does not contribute to CFTR folding, but instead increases the length of time that CFTR remains in the ER, resulting in increased CFTR turnover [98].

### 5.2. E3 Ubiquitin Ligases and Protein Degradation

The complex quality control mechanisms responsible for CFTR folding, maturation, and processing result in a high rate of protein turnover, even for wtCFTR. In fact, current estimates indicate that only one fourth of wtCFTR is folded correctly and trafficked from the ER, whereas virtually no Phe508del-CFTR manages to escape [99]. Proteins unreleased from the ER enter the ubiquitin-proteasome ERAD pathway [34]. Proteins targeted for ERAD by chaperones like CHIP and RMA1 (discussed in *Heat-shock proteins*) are tagged with ubiquitin, a 76-amino acid polypeptide that signals the release of CFTR from the ER membrane. However, rather than trafficking to the cell membrane, ubiquitin-tagged CFTR is hydrolyzed by the proteolytic chymotrypsin-like activity of the proteasome [100].

The first step of the ubiquitin-proteasome ERAD pathway requires the ATP-dependent binding of ubiquitin to an E1 ubiquitin activating enzyme. The E1 enzyme catalyzes the C-terminus of ubiquitin and then transfers ubiquitin to an E1 active site cysteine residue. UBA1 and UBA6 are the only known E1 ubiquitin activating enzymes in humans [101]. Next, E2 ubiquitin-conjugating enzymes are recruited to E1-ubiquitin complex and catalyze the transfer of ubiquitin to the active site cysteine of E2. Currently, 35 unique E2 conjugating enzymes have been identified in the human genome [102]. Lastly, E3 ubiquitin ligases function as substrate identification molecules and bind both E2 enzymes and substrates while transferring ubiquitin from the E2 to the substrate. E3 ubiquitin ligases often transfer multiple ubiquitin polypeptides to a substrate, creating a polyubiquitin chain. Ubiquitinated proteins are then trafficked to the proteasome for degradation. E3 ubiquitin ligases have been sorted into multiple classes, including HECT and RING, depending on their active domains. Over 600 unique E3 ubiquitin ligases have been identified thus far, and most E3s can target multiple substrates. Likewise, individual substrates may be ubiquitinated by multiple E3s [103,104].

To date, several E3 ubiquitin ligases have been shown to ubiquitinate CFTR. RMA1 (sometimes called RNF5) and RNF185, a highly conserved homologue of RMA1, ubiquitinate misfolded CFTR following NBD1 translation [90,105,106]. An additional E3 ubiquitin ligase, gp78, acts by elongating the polyubiquitin chains initiated by RMA1 and RNF185 [107]. Unlike RMA1 and RNF185, the E3 ubiquitin ligase CHIP only acts on fully translated CFTR [78,79,86]. As RMA1, RNF185, and CHIP are unable to directly bind to CFTR, these proteins ubiquitinate misfolded CFTR through adaptor proteins. Specifically, RMA1 and RNF185 require Derlin-1, whereas CHIP binds Hsc70 or Hsp70 [108,109,110]. Interestingly, whereas Hsc70/Hsp70 often promote CFTR folding, CHIP is able to “hijack” these chaperones to trigger ERAD [111]. CHIP and E3 ubiquitin ligase RFFL can ubiquitinate CFTR at the cell periphery. Unlike CHIP, RFFL binds directly to CFTR and is independent of molecular chaperones. RFFL does not affect turnover of wtCFTR, but instead targets only misfolded protein on the cell surface [112].

Additional E3 ubiquitin enzymes that target CFTR include MARCH2, NEDD4-2, SYVN1, and FBXO2. MARCH2 ubiquitinates CFTR through adaptor proteins CAL and STX6 [113]. NEDD4-2 is a HECT E3 ubiquitin ligase that binds both wtCFTR and Phe508del-CFTR [114]. SYVN1 regulates CFTR ubiquitination through the RNF5/AMFR pathway, whereas FBX02 binds directly to CFTR via the SCF complex [115]. Knockdown of MARCH2, NEDD4-2, and SYVN1 has been demonstrated to improve Phe508del-CFTR maturation and trafficking and restore partial function to the mutant protein. Lastly, the deubiquitinase USP10 has been shown to interact with wtCFTR in endosomes, reducing polyubiquitination and improving rates of endocytic recycling of wtCFTR [116,117].

### 5.3. ER Stress and Anterograde Trafficking

Whereas properly folded CFTR usually enters COPII-coated vesicles budding from the ER and subsequently trafficks to the Golgi en route to the cell surface, mutant CFTR can also undergo unconventional anterograde trafficking. Such trafficking is commonly induced by ER stress and involves the bypassing of the ER-to-Golgi transport, resulting in CFTR trafficking from the ER straight to the cell periphery [118,119]. During ER stress, IRE1 initiates the unfolded protein response (UPR), which increases both the expression of Sec16a and the number of possible exit sites in the ER. Sec16a acts as a secretory protein at such exit sites and facilitates scaffolding of COPII-coated vesicles. ER stress also causes GRASP55, usually found in the Golgi, to traffic to the ER, where it interacts with Sec16a. Although the mechanism by which GRASP55/Sec16a aids in the trafficking of CFTR to the cell surface is not currently understood, the resultant membrane bound CFTR lacks complex glycosylation, indicating that the protein bypassed the Golgi [119,120,121]. Despite being misfolded and incompletely glycosylated, Phe508del-CFTR protein that reaches the cell surface through unconventional anterograde trafficking retains partial function, suggesting that the GRASP55 pathway may serve as an interesting therapeutic target.

### 5.4. Protein Kinases and Membrane Stability

Membrane stability of CFTR is partially regulated by protein kinases. PKA and protein kinase C (PKC) have been shown to phosphorylate CFTR predominately at the R domain, although NBD1 and C-terminal residues can also be phosphorylated [122,123,124]. While phosphorylation generally suppresses endocytosis, AMP-activated protein kinase (AMPK) and spleen tyrosine kinase (SYK) have been shown to decrease CFTR plasma membrane stability [124,125]. In the ER, mixed-lineage kinase 3 (MLK3) is believed to promote degradation by interacting with HOP [126]. Additionally, inhibition of the phosphatidylinositol 3-kinase (PI3K) pathway can increase CFTR stability and expression [127,128].

### 5.5. Tethering Factors and Endocytosis Adaptors

Also contributing to CFTR’s plasma membrane stability are endocytosis adaptors and tethering factors. Knockdown of the endocytosis factor DAB2 has been shown to stabilize Phe508del-CFTR at the cell surface by inhibiting endocytosis [129,130]. CFTR has a PDZ binding motif at the C-terminus that tethers to the PDZ domain of NHERF1, supporting channel activation and CFTR membrane stability for both wild-type and mutant proteins [131]. The exchange protein EPAC1 strengthens this interaction and further suppresses endocytosis [132]. However, the CFTR-associated ligand (CAL) decreases the stability of CFTR at the cell membrane through its PDZ domain. Knockdown of CAL has been shown to improve function and stability of Phe508del-CFTR, suggesting that inhibition of the protein may be a therapeutic option [113,133,134].

## 6. Small Molecule Modulators

The observation by Welsh and colleagues that Phe508del-CFTR could be rescued and traffic to the cell surface via low temperature (27° C) incubation was transformative because it demonstrated that if Phe508del (and potentially other mutations) could escape the ERAD pathway and traffic to the cell membrane, they retained partial function [31]. While low temperature treatment is not a therapeutically viable option for CF patients, this observation encouraged researchers to target genes affecting the processing and maturation of CFTR [135]. Furthermore, through the use of high-throughput screening technology, several small molecules that interact directly with CFTR and positively affect processing or function have been identified. Following lead optimization and clinical trials, four small molecules are now FDA-approved for CF, providing potentially 90% of patients with at least one modulator option. Several other small molecules are currently being investigated in clinical trials (Table 1).

### 6.1. CFTR Potentiators

Potentiators are a class of small molecules that increase anion transport via CFTR at the cell membrane by increasing the channel open probability. As discussed previously, Class III CFTR mutations have gating defects, whereas Class IV CFTR mutations exhibit abnormal conductance. However, in both mutation classes, CFTR is trafficked to the cell membrane and partial function can be restored with the use of potentiators. Furthermore, clinical benefits have been observed in patients with G551D and related mutations when receiving monotherapy with a single potentiator [136,137,138,139,140,141]. As will be discussed later, potentiators can also help to restore function in Class II mutations, including Phe508del-CFTR, when coupled with one or more correctors.

Vertex Pharmaceuticals (Boston, MA, USA) identified the first FDA-approved CFTR potentiator. Ivacaftor (trade name Kalydeco) was approved in 2012 for CF patients with the G551D mutation and was later approved for additional Class III mutations, including G1244E, G1349D, S549R, among others [140,142,143,144,145,146]. Kalydeco has also been approved for the Class IV mutation R117H and related mutations [141,147,148]. Ivacaftor was identified through a high throughput screen using NIH-3T3 mouse fibroblast cells expressing Phe508del-CFTR [149]. These cells were first incubated at 27 °C to rescue the mutant protein to the cell surface and then treated with small molecule candidates, with a fluorescent signal being detected when CFTR-mediated chloride transport occurred. Vertex screened approximately 300,000 compounds using the NIH-3T3 fluorescence assay and identified four scaffolds that had significant potentiating activity. Following medicinal chemistry to optimize these scaffolds, investigators concluded that a singular scaffold was the most efficacious and lead optimization of the scaffold resulted in the testing of an additional 70 small molecules. Of these, VX-770, which would later be named ivacaftor, showed superior function. In human bronchial epithelial (HBE) cells derived from G551D/Phe508del subjects, ivacaftor increased chloride secretion 10-fold, reaching 50% of wtCFTR levels [136]. In vivo studies in G551D patients followed, and two randomized, double-blind, placebo-controlled studies demonstrated a 10.5% increase in FEV_1_ compared to placebo and markedly reduced sweat chloride levels [143]. These groundbreaking results were the first in which a small molecule acted as a clinical modulator of CFTR. Patch clamp studies of ivacaftor-treated cells expressing G551D concluded that ivacaftor increased the open probability of G551D-CFTR six-fold [136]. Additionally, ivacaftor increased the open probability of Phe508del-CFTR five-fold and even wtCFTR two-fold [136,139,150]. Further clinical trials of ivacaftor in Phe508del patients did not yield efficacious results [151].

The flavonoids genistein and curcumin have also been shown to have potentiating effects, especially when combined with lumacaftor (see CFTR Correctors), as these compounds enhance forskolin-induced swelling in rectal organoids with Phe508del and G551D mutations [152]. Rattlesnake phospholipase A_2_ and several aminoarylthiazoles are also being investigated as potentiators in CFBE41o- and Phe508del-A549 cells [153,154,155]. Furthermore, several pharmaceutical companies and research groups have small molecule potentiators in clinical trials, including Vertex, AbbVie, and Proteostasis Therapeutics (Table 1).

### 6.2. CFTR Correctors

While potentiators like ivacaftor have had significant clinical benefits for patients with Class III and Class IV mutations, little effect was seen in patients with Class II mutations, including those with Phe508del. As approximately 90% of CF patients have at least one Phe508del allele, there is substantial interest in identifying small molecule correctors that can restore function to misfolded proteins with processing defects.

Corr-4a was the first corrector discovered that restored function to Phe508del-CFTR-transfected epithelial cells at 37 °C to the same level as low temperature incubation [156]. High throughput screening has aided researchers in the quest to discover small molecule correctors and such experiments led Vertex Pharmaceuticals to discover VRT-422 and VRT-325 [137]. These compounds restored CFTR-mediated chloride conductance to 10% of wtCFTR levels in HBE cells. Despite this modest restoration of conductance, these compounds served as an important proof-of-concept for small molecule correction at a clinically relevant level. Further medicinal chemistry and lead optimization by Vertex led to the discovery of VX-809, which reportedly rescued up to 30% of Phe508del-CFTR from degradation and restored chloride conductance to approximately 15% of wtCFTR levels [157]. VX-809 was later named lumacaftor. Interestingly, lumacaftor shows added efficacy when combined with low temperature, Corr-4a, and VRT-325, indicating that the misfolding defect(s) caused by the Phe508del-CFTR mutation is not entirely corrected by the individual compound [158,159,160]. This strongly supports a therapeutic strategy of combining more than one corrector compound.

Following the identification of lumacaftor and ivacaftor, the corrector-potentiator combination entered clinical trials. Once approved for Phe508del homozygous patients, the combination, marketed as Orkambi, gave up to 45% of CF patients a small molecule modulator option [161]. However, the clinical effect seen in Phe508del patients treated with Orkambi was modest, as homozygous patients experienced an average increase in FEV_1_ of only 4% [162]. Additionally, an antagonistic effect between ivacaftor and lumacaftor was seen in several studies, leaving many patients without clinical improvement [163,164]. An estimated 15% of patients discontinued Orkambi within three months of use [165].

Recognizing the need for improved correctors, as well as the potential benefits of multi-corrector treatments, Vertex developed VX-661, later named tezacaftor, and VX-445, renamed elexacaftor. While tezacaftor is structurally related to and shares a mechanism with lumacaftor, elexacaftor is thought to act at a second site on CFTR, making it a corrector 2 (or C2) molecule [166]. Clinical trials with a triple-combination of tezacaftor, elexacaftor, and ivacaftor in patients with at least one Phe508del allele resulted in an average increase in FEV_1_ of approximately 10%, as well as reduced sweat chloride and frequency of pulmonary exacerbations [167,168]. This triple-combination therapy was subsequently FDA-approved in 2019 for patients with at least one Phe508del allele and marketed as Trikafta.

Vertex has also completed clinical trials of VX-440 and VX-152 in combination with tezacaftor/ivacaftor, but pursued elexacaftor as the third element of their triple-combination strategy [169]. Additional correctors in clinical trials have been reported by Vertex, AbbVie, Flatley Discovery Lab, and Proteostasis Therapeutics (Table 1) [170,171].

### 6.3. Premature Stop Codon Readthrough Agents

Although Kalydeco and Trikafta provide up to 90% of CF patients with modulator treatments, such small molecules are not therapeutic for patients with Class I mutations, which cause unstable mRNA and often no protein production. An estimated 9% of CF-causing mutations fall in Class I and approximately half of all Israeli CF patients have such mutations [172,173]. As most Class I mutations are caused by a premature stop codon, “readthrough” of these stop codons would theoretically allow for proper translation to the normal transcript termination site. This effect has been seen in R553X- and G542X-CFTR-expressing HeLa cells treated with aminoglycoside antibiotics, such as gentamicin [174,175]. However, preclinical studies of gentamicin treatment in patients with Class I mutations showed no clinical benefit [176,177].

High-throughput screens identified ataluren as a potentially efficacious readthrough agent. In subsequent experiments in transgenic mice harboring the G542X mutation, CFTR expression at the plasma membrane was partially restored by ataluren treatment [178,179,180]. However, while ataluren progressed to phase III clinical trials, little benefit was observed [181]. Currently, synthetic aminoglycosides, ataluren derivatives, and escin, the FDA-approved active component of horse chestnut seed, are being investigated as readthrough agents in W1282X/Phe508del-CFBE and human primary epithelial cells [182,183,184,185]. It is important to note that such agents may cause the insertion of non-native amino acids at the site of readthrough, which may reduce channel function [186]. Currently, ELX-02, a eukaryotic ribosomal selective glycoside developed by Eloxx Pharmaceuticals (Waltham, MA, USA), is in Phase 2 clinical trials as a premature stop codon readthrough agent (NCT04135495).

### 6.4. CFTR Stabilizers

While CFTR correctors can rescue mutant protein to the cell surface, long-term stability of Phe508del-CFTR at the plasma membrane has not been observed following solo corrector treatment [187]. Likewise, while low temperature treatment rescues CFTR to the cell surface, the protein’s half-life is still reduced and it experiences increased endocytosis and decreased recycling [188,189]. Class VI CFTR mutations result in unstable protein configurations that lead to reduced residency of CFTR at the cell surface and, therefore, less anion conductance. As correctors are currently unable to address this class of mutations, researchers have searched for small molecules to stabilize the mutant CFTR protein at the plasma membrane for longer periods.

To date, several CFTR stabilizers have been identified. Although not a small molecule, hepatocyte growth factor (HGF) has been shown to activate Rac1 signaling and resultingly stabilize CFTR through its interaction with NHERF-1 [190]. While lumacaftor can increase CFTR plasma membrane stability to a modest degree, co-treatment of lumacaftor with HGF further enhanced the anchoring of CFTR to NHERF-1 in mouse small intestine organoids [131,191]. Treatment with vasoactive intestinal peptide also stabilized interactions between CFTR and NHERF-1 by decreasing the rate of endocytosis [192]. Lastly, cavosonstat, an inhibitor of S-nitrosoglutathione reductase, helps to stabilize CFTR by preventing its interaction with HOP [76,77]. Interestingly, cavosonstat is the only CFTR stabilizer to be tested in clinical trials (NCT02589236). It is currently being administered to Phe508del/Phe508del patients using Orkambi and patients with Class III mutations using Kalydeco.

### 6.5. Splicing Correctors

Approximately 10% of CFTR mutations are caused by aberrant mRNA splicing that often results in immature protein that rarely trafficks to the cell membrane. Such mutations can be found across multiple classes but are particularly common in Class V. Modulators able to correct splicing and restore full-length CFTR mRNA could rescue CFTR protein function. Currently, antisense oligonucleotides are being investigated as therapeutic options for splicing mutations [193].

### 6.6. CFTR Amplifiers

Amplifiers increase the amount of *CFTR* mRNA production and subsequent protein production [194]. As the mRNA still contains a mutation, amplifiers do not directly correct processing or restore function to the protein. Instead, an increased amount of protein substrate is available for modulators to act upon. Therefore, amplifiers are always investigated as a component of a multi-drug therapy. Phase 2 clinical trials were recently completed for the amplifier PTI-428, or nesolifcaftor, in patients using tezacaftor/ivacaftor (NTC03591094).

### 6.7. mRNA Delivery Agents

Delivery of *CFTR*-encoding mRNA to the lungs would allow epithelial cells to create wtCFTR protein in a mutation-agnostic manner. Robinson and colleagues used lipid-based nanoparticles (LNPs) for delivery of chemically modified *CFTR* mRNA (cmCFTR) to CFTR knockout mice [195]. Approximately 55% of net chloride efflux of normal mice was observed 3 days post-transfection. Translate Bio is currently testing MRT5005, an agent designed to deliver *CFTR* mRNA, in Phase I clinical trials (NCT03375047).

### 6.8. Proteostasis Modulators

Glycerol and trimethylamine N-oxide (TMAO), when added to NIH 3T3 cells expressing Phe508del, were found to restore partial processing and function to Phe508del-CFTR [196,197,198]. High concentrations of 4-phenylbutrate (4PBA) emerged as a candidate CFTR modulator, as it restored function to Phe508del-CFTR by interfering with Hsc70 in HEK293 cells expressing Phe508del. However, clinical trials of 4PBA showed little improvement in respiratory function [199,200,201,202]. Balch and colleagues tested HDAC inhibitors for the rescue of Phe508del-CFTR and identified suberoylanilide hydroxamic acid (SAHA) as efficacious in primary human bronchial epithelial cells [203,204]. The combination treatment of cysteamine and epigallocatechin gallate has been shown to rescue CFTR trafficking, function, and plasma membrane stability through the correction of Beclin-1 autophagy flux in primary nasal epithelial cells [205,206]. A phase II clinical trial of the cysteamine-epigallocatechin gallate combination reported decreased sweat chloride levels and modest increases in FEV_1_ in Phe508del/Phe508del patients [207].

McCray and colleagues used a transcriptomic-based strategy to identify candidate correctors of CFTR. By querying the genomic signature of miR-138-mediated CFTR rescue in the Connectivity Map, a catalogue of gene expression profiles of various cell lines treated with bioactive small molecules, the group was able to identify molecules whose genomic signatures closely resembled that of miR-138 overexpression or *SIN3A* knockdown. After testing 27 small molecules, four were identified that partially rescued maturation and function of Phe508del-CFTR in primary human airway epithelia (HAE), including biperiden, pizotifen, pyridostigmine, and valproic acid. Of these, pyridostigmine showed cooperativity with corrector compound C18 (an analogue of lumacaftor) in improving Phe508del-CFTR function [208].

Likewise, Galietta and colleagues used connectivity mapping to identify drugs having a similar mode of action at the gene expression level as CFBE41o- and primary bronchial epithelial cells treated at 27 °C for 24 h [209]. Several anti-inflammatory glucocorticoids were found to increase Phe508del-CFTR function in the cell line, but the activity could not be confirmed in primary cells. Sondo et al. also identified 9-aminoacridine and ciclopirox as proteostasis regulators able to restore partial function to Phe508del-CFTR in cell lines [210]. However, these small molecules did not increase chloride secretion in primary bronchial epithelial cells from CF patients and subsequent microarray profiling revealed different gene expression signatures generated by the treatments in cell lines and primary cells.

Additional investigations of the repurposing of drugs currently FDA-approved for non-CF disorders yielded compounds that are efficacious in vitro. Miglustat (marketed under the trade name Zavesca and used to treat Gaucher disease) and sildenafil (marketed under the trade name Viagara and used to treat erectile dysfunction and pulmonary hypertension) treatments partially restored function to Phe508del-CFTR in human nasal epithelial cells [211,212].

## 7. Conclusions

A golden age of CFTR small molecule modulators has arrived, as approximately 90% of CF patients could receive clinical benefits from the use of one or more FDA-approved drugs. Clinical studies have reported improved lung function, reduced pulmonary exacerbations, increased weight, and improved quality of life measures. However, despite the profound impact that these drugs are having on patients, there are major areas that must be considered in the future of CF drug development. First, it is crucial that therapies be identified for all CFTR mutation classes. Currently, there are no approved treatments for mutations causing premature stop codons, frameshifts, or nonsense mutations. Fortunately, small molecules to address some of these mutations are currently progressing through clinical trials. However, it is possible that small molecule therapeutics will not provide clinical benefits to all mutations. For such situations, the development of gene therapy or gene editing approaches may be crucial [213,214]. Secondly, there are still patients in age ranges that are not approved to receive the FDA-approved small molecules. CFTR modulators are likely to have their greatest benefit if patients are treated before irreversible tissue remodeling of the lung occurs, presumably shortly after birth, or even *in utero*. Orkambi is currently being tested in patients 12-24 months old, whereas Trikafta is under investigation in patients 6-11 years of age. Lastly, the long-term effects of the current treatments are unknown, as the drugs only recently became available. Additionally, they are currently quite expensive, which limits their widespread availability worldwide, and places burdens on healthcare systems. It will be important for healthcare professionals to continually monitor the efficacy and any potential side effects of these compounds.

While the advancements in small molecule treatments for CF in the last decade have been monumental and are acknowledged, it is imperative that improved treatments continue to be developed. As seen with Trikafta, the co-treatment of two or more small molecules may present opportunities to improve the efficacy of pharmacological therapies. Tezacaftor and elexacaftor act synergistically, likely due to the fact that elexacator acts on a different site in CFTR than tezacaftor. An alternative strategy is to pair a corrector that interacts directly with CFTR, such as tezacaftor, which a small molecule that manipulates the CFTR proteostasis pathway. Significant additive effects have been observed in human primary airway epithelial co-treated with pyridostigmine and corrector compound C18, as well as in SAHA paired with corrector compound C3. While these small molecules have not yet advanced to clinical trials, they lend support to the strategy of targeting proteostasis interactors. Furthermore, although several modulators of CFTR proteostasis have not shown significant efficacy in clinical trials, it would be unwise to abandon investigations of such therapies. Some failed candidates, such as glycerol and 9-aminoacridine, have incompletely understood mechanisms, making lead optimization difficult. Others, such as 4PBA, target proteins that can act in both degradation and maturation pathways, further complicating the already delicate process of rescuing Phe508del. However, as seen in Figure 4, many proteins involved in the CFTR proteostasis pathway have not been targeted pharmaceutically for correction of Phe508del and may be therapeutic targets. Lastly, as transcriptomic and proteomic studies of CFTR proteostasis continue to uncover new interactors, it is important for researchers to investigate whether such interactors can be targeted therapeutically. Modulators of the CFTR proteostasis pathway could serve as pharmaceutical leads and complement the already existing drugs discovered via high throughput screening.

## Figures and Tables

**Table 1:** Selected clinical trials using small molecule modulators for cystic fibrosis.

Small molecule(s)	Trade Name	Company	Phase	Modulator Type	Mutations	Clinical Trial
Ivacaftor	Kalydeco	Vertex Pharmaceuticals	Approved	Potentiator	G551D, 37 others *	NCT02725567
Lumacaftor + Ivacaftor	Orkambi	Vertex Pharmaceuticals	Approved	Lumacaftor = Corrector; Ivacaftor = Potentiator	Phe508del/Phe508del	NCT03601637
Tezacaftor + Ivacaftor	Symdeko	Vertex Pharmaceuticals	Approved	Tezacaftor = Corrector; Ivacaftor = Potentiator	Phe508del/Phe508del,26 others **	NCT02412111
Elexacaftor + Tezacaftor + Ivacaftor	Trikafta	Vertex Pharmaceuticals	Approved	Elexacaftor, Tezacaftor = Correctors; Ivacaftor = Potentiator	Phe508del + any other mutation	NCT04183790
PTC124 + Ivacaftor	Ataluren	PTC Therapeutics	Phase 4	Premature stop codon readthrough	Class I mutations	NCT03256968
VX-561	---	Vertex Pharmaceuticals	Phase 2	Potentiator	G551D, 8 others ***	NCT03911713
ABBV-2222	---	AbbVie	Phase 2	Corrector	Phe508del/Phe508del	NCT03969888
ABBV-3067	---	AbbVie	Phase 2	Potentiator	Phe508del/Phe508del	NCT03969888
ELX-02	---	Eloxx Pharmaceuticals	Phase 2	Nonsense mutation readthrough agent	G542X	NCT04135495
FDL169	---	Flatley Discovery Lab	Phase 2	Corrector	Phe508del/Phe508del	NCT02767297
PTI-428 + Ivacaftor	---	Proteostasis Therapeutics	Phase 2	Amplifier	Same as Ivacaftor	NCT03258424
PTI-801	---	Proteostasis Therapeutics	Phase 2	Corrector	Phe508del/Phe508del	NCT03140527
PTI-808	---	Proteostasis Therapeutics	Phase 2	Potentiator	Phe508del/Phe508del	NCT03251092
VX-121	---	Vertex Pharmaceuticals	Phase 2	Corrector	Phe508del + minimal function (MF) mutation	NCT03912233
MRT5005	---	Translate Bio	Phase 1	mRNA delivery	Class I or II mutations	NCT03375047

* Mutations *A455E, A1067T, D100E, D110H, D579G, D1152H, D1270N, E56K, E193K, E831X, F1052V, F1074L, G178R, G551S, G1069R, G1244E, G1349D, K1060T, L206W, P67L, R74W, R117C, R347H, R352Q, R1070Q, R1070W, S549N, S549R, S945L, S977F, S1251N, S1255P, R117H, c.579+3A>G, c.2657+5G>A, c.3140-26A>G, c.3717+12191C>T*. ** Mutations *A455E, A1067T, D100E, D110H, D579G, D1152H, D1270N, E56K, E193K, E831X, F1052V, F1074L, K1060T, L206W, P67L, R74W, R117C, R347H, R352Q, R1070W, S945L, S977F, c.579+3A>G, c.2657+5G>A, c.3140-26A>G, c.3717+12191C>T*. *** *G178R, G551S, G1244E, G1349D, S549N, S549R, S1251N, S1255P*.
